# Correction: PPM-Decay: A computational model of auditory prediction with memory decay

**DOI:** 10.1371/journal.pcbi.1008995

**Published:** 2021-05-26

**Authors:** Peter M. C. Harrison, Roberta Bianco, Maria Chait, Marcus T. Pearce

A grant acknowledgement was missing from the funding statement. The funding statement for this article should read as follows:

“PH was supported by a doctoral studentship from the Engineering and Physical Sciences Research Council (EPSRC, https://epsrc.ukri.org/) and Arts and Humanities Research Council (AHRC, https://ahrc.ukri.org/) Centre for Doctoral Training in Media and Arts Technology (EP/L01632X/1). The research was additionally funded by a BBSRC grant (BB/P003745/1) to MC and supported by the NIHR UCLH BRC Deafness and Hearing Problems Theme. The funders did not play any role in the study design, data collection and analysis, decision to publish, or preparation of the manuscript.”
