# Supplementary material for: PPM-Decay: A computational model of auditory prediction with memory decay
Source: PLoS Comput Biol. 2020 Nov 4;16(11):e1008304. doi: 10.1371/journal.pcbi.1008304 (PMC7668605; doi:10.1371/journal.pcbi.1008304)
Supplement: Attachment — Submitted filename: response to reviewers.pdf [file pcbi.1008304.s001.pdf]

Response to Editor

Editor comment:

Both reviewers agreed that your work has merit but 1) many points need clarification and 2) it might be less novel than advertised. You will want to try the models suggested by reviewer 1 and broaden your discussion to provide fair comparisons with the computational methods used in linguistics.

Author response:

We are very grateful for the detailed comments provided by the reviewers on our manuscript. We have systematically addressed these comments, resulting in what we think is a significantly improved manuscript. In particular, many points have been clarified concerning the relationship of the present work to existing literature in psychology and computational linguistics, helping to delineate which aspects of the present work are novel and which have precedents in previous work. We have also referred to reader to some additional model comparisons as suggested by Reviewer 1.

Response to Reviewer 1

Reviewer comment:

The authors present essentially an n-gram model that more heavily weights recent data and show that it out-performs an unweighted model on a simple toy dataset, and then that it does a better job of predicting note sequences in a music dataset. Then, the authors model experimental data in which participants listen to tone sequence and must detect a shift to randomness. They compare a variety of different versions of this model, including PPM, PPM+decay, and PPM+decay+retrieval noise with varying item buffers, and find that models with retrieval noise are a substantially better fit to human performance.

The results and model seem reasonable, but there is a huge literature on related methods, mostly in computational linguistics, that seem worth connecting to in some way. For instance, there are many Hidden Markov Model inference schemes that take observations of discrete sequences and try to construct a markov model to make predictions, and in this inference there are often naturally arising forms of memory decay when making predictions. There is also related work on clustering input sequence (for a historical reference, see Carter "Improving Language Models by Clustering Training Sentences").

Finally, there are versions of this kind of decayed n-gram model sometimes under the name of "language model adaptation" (I don't know all of them, but a start in the literature are

"Language model adaptation using mixtures and an exponentially decaying cache", "Time-Sensitive Language Modelling for Online Term Recurrence Prediction", and "Statistical language model adaptation: review and perspectives")

I think that before I could recommend acceptance, the work would have to be brought in contact with this broader literature. I don't think the existence of these kinds of models really impedes the authors' ability to publish, I just would not want them to miss the opportunity to connect to a broader modeling literature.

Author response:

We're happy to have been pointed to this related work in the computational linguistics literature, and have now explored these and related papers. In the Introduction, we have added a paragraph reviewing decay models within the context of model adaptation. For balance, we have also added a paragraph reviewing cognitive sequence models that incorporate explicit or implicit decay effects. In the Discussion, we have added a paragraph describing the clustering approach to model adaptation, and how it might profitably be incorporated into the PPM-Decay model.

The exponential decay property of the standard hidden Markov model is interesting. Strictly speaking, though, this decay phenomenon is not directly comparable to the decay models described here: in the hidden Markov model, the exponential decay concerns the mutual information between the next Markov state and a historic Markov state, rather than the mutual information between the next Markov state and historic training data. We have therefore not discussed the exponential-decay property of the standard hidden Markov model in the main manuscript because we feel it might muddy the waters; however, we have now mentioned a variant of the hidden Markov model with explicit exponential decay in the training function, which we feel provides a better analogy to the PPM-Decay model.

Revised text:

[Introduction, l. 98-123]:

Some sequence modeling approaches from the cognitive literature do incorporate phenomena such as recency effects and capacity limits. Squires et al. (1976) describe a hand-crafted model for expectations elicited by symbolic sequences that incorporates an exponential-decay component. O'Reilly (2013) and Skerritt-Davis and Elhilali (2019) describe Bayesian inference approaches where continuous observations are assumed to be generated from Gaussian distributions whose underlying parameters change at unknown points, a situation where the optimal inference strategy involves downweighting historic observations. The latter paper's model additionally incorporates a fixed bound on memory capacity and a perceptual noise parameter, improving its cognitive plausibility. Meyniel et al. (2016) describe a similar approach with categorical observations generated by a first-order Markov model, and Broker et al. (2018)

model similar data using both a hierarchical Bayesian model and a simpler exponential-decay model. Mattar et al. (2016) present participants with sequences of synthetic face images, and model resulting brain activity with an exponential-decay memory model. These studies demonstrate the importance of recency effects for sequence modeling; however, the resulting models generally cannot learn the kinds of higher-order statistics that PPM specializes in.

Some approaches from the natural language processing literature also incorporate recency effects. Here the motivation is primarily 'model adaptation', helping the model to respond to changing statistics in the data being modeled; a useful byproduct can be reducing the computational resources required by the implementation. A recency effect with a particularly efficient implementation is exponential decay, which has been applied to a variety of model types, such as trigram word models (Clarkson & Robinson, 1997; Osborne et al., 2014), Web search term recurrence models (Zhang et al., 2009), topic models (Mikolov & Zweig, 2012), data streaming models (Cormode et al., 2009), and hidden Markov models (Stenger et al., 2001). Related heuristics are the sliding window of the Lempel-Ziv data compressor (Ziv & Lempel, 1977) and the nonstationary update rule of the PAQ compressor (Mahoney, 2005). However, these models have yet to be integrated into mainstream cognitive modeling research, having been primarily optimized for engineering applications rather than for cognitive plausibility.

[Discussion, l. 733-745]

The current PPM-Decay model uses memory decay to cope with changes in sequence statistics. This technique works well if sequence statistics are always changing to new, unfamiliar values; however, in the natural world it is quite possible that historic sequence statistics end up returning in the future. In such scenarios, it would be desirable to have retained a memory of these historic statistics, and to reactivate this memory once it becomes relevant again. It seems plausible that humans do something like this; for example, individuals who learn multiple languages seem able to learn separate models for each language, and switch between these models according to context. Something similar may happen when individuals become familiar with multiple musical styles (Huron, 2006). In the domain of natural language processing, various techniques have been developed that implement such a model switching process, with input sequences being allocated to models using either prespecified assignments or unsupervised clustering (e.g. Bellegarda, 2004; Kneser & Steinbiss, 1994; Kneser & Peters, 1997; Martin et al., 1997). It would be worthwhile to incorporate similar techniques into the PPM-Decay model.

***

Reviewer comment:

- It strikes me that PPM-decay shares a lot of similarities with Lempel-Ziv compression (or any variants, like LZW) in that repeated data is given a shorter code word. I think it would strengthen the paper substantially to test out some of these other compression schemes to be

sure that PPM-decay is not just doing some kind of approximation scheme to another compression scheme.

Author response:

We agree - the PPM algorithm and the Lempel-Ziv algorithm do share a lot of similarities. Begleiter et al. (2004, *On Prediction Using Variable Order Markov Models,* https://arxiv.org/pdf/1107.0051.pdf) provide a nice explanation of how the Lempel-Ziv algorithm can be formulated as an incremental sequence prediction model, and it can be clearly seen from this explanation how Lempel-Ziv provides a simplified approximation to PPM without PPM's sophisticated smoothing mechanism. As suggested by the reviewer, it makes sense to motivate the choice of PPM over Lempel-Ziv through a performance comparison; fortunately such comparisons already exist in the literature, and we have added some pointers to those comparisons to the Discussion. We have also broadened the discussion to other competing models from the literature.

Revised text:

[Discussion, l. 782-812]

We chose to focus on extending PPM for the present work because PPM already plays an important role in the predictive processing literature. However, there are many other algorithms that could theoretically be used in its place. When choosing a potential replacement for PPM, it is important to bear three principles in mind: a) how well the algorithm approximates an ideal observer, as quantified by corpus modeling; b) how plausible the algorithm would be as a cognitive model; c) how easily the algorithm can be customized to implement cognitively motivated features. One prominent competitor from the data-compression literature is the Lempel-Ziv algorithm (Ziv & Lempel, 1978), an example of a dictionary-coding model that can be formulated as an incremental sequence prediction model like PPM (Langdon, 1983; Rissanen, 1983; Begleiter et al., 2004). Formulated this way, Lempel-Ziv has a similar form to PPM, but one that trades PPM's complex blending strategies for increased computational efficiency. Consequently, PPM systematically outperforms Lempel-Ziv algorithms on data compression benchmarks (Bunton, 1996; Begleiter et al., 2004; Bell et al., 1990); we therefore prefer PPM for our purposes, as it provides a better approximation to an ideal observer. A second prominent competitor from the data-compression literature is the PAQ family of algorithms, which takes the *n*-gram blending principle of PPM as a starting point and adds many additional features, such as neural networks for combining sub-models, two-dimensional contexts for modeling spreadsheets, and specialized models for BMP, TIFF, and JPEG image formats (Mahoney, 2013). While the PAQ algorithms outperform PPM on standard data compression benchmarks (Mahoney, 2005), their specialization to computer file formats makes them implausible as cognitive models. A third potential competitor from the machine-learning literature is the recurrent neural network, a model that (given sufficiently many parameters and appropriate training) has a powerful universal approximator property (Schäfer & Zimmermann,

2006). However, such networks typically require a lot of training data to reach high performance levels, and it is difficult to see how they could incorporate fully customizable memory decay functions (though some kinds of memory decay could be achieved through a combination of online learning and regularization). In conclusion, we chose to use PPM as it provides an effective balance of predictive power, cognitive plausibility, and customizability that makes it well-suited to modeling auditory predictive processing; nonetheless, future research might profitably consider potential alternative models, and seek to collect cognitive data that can help choose between such models.

***

Reviewer comment:

- Exponential weighting is interesting, but language (and probably music) follow a power-law time series dependency. The authors might be interested in Anderson & Schooler "Reflections of the environment in memory" who look at the gap between word repetitions and show they are power-law distributed. It seems like a similar empirical approach to quantify the distribution of repetitions of subsequences could be useful here, and I'd be surprised if it wasn't power law.

Author response:

This is an interesting point. We have added some text to the Introduction noting how exponential kernels are typically favored for their efficient computational implementation, which complements pre-existing text in the Discussion discussing the computational efficiency of exponential kernels and the consequent potential for improving the efficiency of the PPM-Decay model. Further, we have added a paragraph to the Discussion discussing power-law relationships in music and language, and how the PPM-Decay model could be adjusted to reflect or test these relationships.

Revised text:

[See above for changes to Introduction, l. 116-121]

[Discussion, l. 771-781]

We modeled musical corpora using PPM-Decay with an exponentially decaying memory kernel. However, it has been argued that sequential dependencies in many natural sequences (e.g. language, birdsong) have power-law decay, not exponential decay (Sainburg et al., 2019; Anderson & Schooler, 1991). To the extent that music is characterized by syntactic expressivity greater than that of a probabilistic regular grammar (e.g. Rohrmeier 2011), we would also expect it to exhibit power-law dependencies (Lin & Tegmark, 2017). It has been argued that human memory also exhibits power-law decay, making it ideally suited to processing natural language (Anderson & Schooler, 1991), though see Anderson (1997; 2001) and Murre (2011) for reasons to

be cautious. A natural follow-up would therefore be to compare PPM-Decay models with exponential decay to equivalent models with power-law decay; if natural sequences and human memory are better characterized by power-law decay, we would expect the latter models to generate more accurate sequence predictions and better simulations of participant behavior.

***

Reviewer comment:

- Why is the PPM-Decay model evaluated based on prediction accuracy instead of a more standard NLP measure like perplexity (2^surprisal)? I like having accuracy since it's intuitive, but I think perplexity is also important to include since it penalizes mistakes differently.

Author response:

We use three evaluation metrics for the model: Experiment 1 uses accuracy, Experiment 2 uses cross-entropy, and Experiment 3 uses reaction time. We explain in the paper how accuracy is a reasonable intuitive measure for Experiment 1, where the alphabet size is small, and how cross-entropy (or equivalently, perplexity) is better for Experiment 2, where the alphabet size is large. Note that cross-entropy is essentially equivalent to perplexity (just removing the 2 ^ component); we have added a note to this effect in the paper.

Revised text:

[Experiment 2, l. 326-333]

Similar to Experiment 1, we supposed that the listener constantly tried to predict the next chord in the chord sequence, but this time we operationalized predictive success using the cross-entropy error metric, defined as the mean negative log probability of each chord symbol as predicted by the model. This metric is more appropriate than mean success rate for domains with large alphabet sizes, such as harmony, because it assigns partial credit when the model predicts the continuation with high but non-maximal probability. A similar metric, perplexity, is commonly used in natural language processing (cross-entropy is equal to the logarithm of perplexity).

***

Reviewer comment:

- The paper needs more details on how the PPM-decay model works -- I only found these in the supplemental. For instance, it would be useful to put some description of the smoothing into the main text.

Author response:

It is a good idea to provide an informal but reasonably detailed overview of the model before describing the experiments; in the original manuscript, the model exposition was left to the Methods section. We have written such an overview now, see below.

Revised text:

[Overview of the PPM-Decay model, l. 158-205]

This section provides a short overview of the PPM-Decay model. A full exposition is provided at the end of the paper.

The PPM-Decay model is a direct extension of the PPM model (Cleary & Witten, 1984) incorporating the interpolated smoothing technique of Bunton (1997) alongside a custom memory retrieval function. PPM is an incremental sequence prediction model: it progresses through sequences symbol by symbol, generating a predictive distribution for the next symbol before it arrives, with this predictive distribution derived from statistics learned from the model's previous observations. The predictive distribution comprises a list of probabilities for all symbols in the alphabet, with likely continuations receiving high probabilities and unlikely continuations receiving low probabilities.

PPM's predictions are produced by combining predictions from several sub-models, specifically *n*-gram models. An *n*-gram is a sequence of *n* adjacent symbols; for example, 'ABCABC' is an example of a 6-gram. An *n*-gram model generates predictions by counting and comparing observations of *n*-grams. To predict what happens after the sequence 'ABCAB', a 6-gram model would look at all 6-gram occurrences of the form 'ABCABX', where 'X' is allowed to vary. The predicted probability of observing 'C' having just observed 'ABCAB' would then be equal to the number of observations for 'ABCABC' divided by the total number of observations for *n*-grams of the form 'ABCABX'.

The *order* of an *n*-gram model is defined as the length of its *n*-grams. Different orders of *n*-gram model have different strengths and weaknesses. Low-order *n*-gram models are quick to learn, but can only capture simple structure. High-order *n*-gram models can capture more complex structure, but require more training data. The power of PPM comes from flexibly switching between different *n*-gram orders based on context; this is why it is termed a 'variable-order' Markov model.

Different PPM variants use different techniques for combining *n*-gram models. The original PPM model used a technique called backoff smoothing, where the model chooses one top-level *n*-gram model from which to generate predictions for a given context, and only calls upon lower-level *n*-gram models in the case of symbols that have never been seen before by that *n*-gram model. Here we instead use the interpolated smoothing technique of Bunton (1997),

where lower-order models contribute to predictions for *all* the symbols in the alphabet. This approach is more computationally expensive, but results in significantly improved predictive power.

The different n-gram models are weighted according to training data availability for the current predictive context: with limited training data, low-order models receive the most weight, but as training data accumulates, high-order models receive increasing weight. The procedure for deriving these weights is termed the 'escape method'; though various improved escape methods have been introduced over the years, here we retain the escape method from the original PPM model (Cleary & Witten, 1984), because it generalizes most naturally to the memory-decay procedures considered here.

The PPM-Decay model adds a custom memory retrieval function to PPM. This retrieval function modifies how n-grams are counted: whereas in the original PPM model, each n-gram observation contributes a fixed amount to the respective n-gram count, in the PPM-Decay model the contribution of a given n-gram observation decreases as a function of the time elapsed since the observation. This function is termed the decay kernel; an example decay kernel is plotted in Fig 1. In the following experiments we explore how decay kernels may be constructed that help predictive performance for particular kinds of sequences, and that reflect the dynamics of human auditory memory.

***

Reviewer comment:

- Why doesn't the model use state-of-the-art smoothing techniques? See, e.g., Chen & Goodman "An empirical study of smoothing techniques for language modeling" is a classic and very good reference on this.

Author response:

We have expanded our explanation in the Methods section of our smoothing methods: basically, the implementations of Kneser-Ney smoothing (and other related techniques such as the use of update-excluded counts in PPM) do not generalize obviously to decay-based models, and in the absence of strong evidence that humans adopt analogous techniques, we thought it best to omit them for now.

Revised text:

[Model, l. 839-846]

Many variants of PPM exist in the literature (Cleary & Witten, 1984; Bunton, 1997; Moffat, 1990; Cleary & Teahan, 1997). Our formulation incorporates the interpolated smoothing technique of

Bunton (1997), but avoids techniques such as Kneser-Ney smoothing, exclusion, update exclusion, and state selection, because it is not obvious how to generalize these techniques to decay-based models where *n*-gram counts can fall between zero and one, and because we do not yet have compelling evidence that human cognition employs analogous methods. Nonetheless, an interesting topic for future work is to explore the cognitive relevance and potential computational implementations of these techniques.

***

Reviewer comment:

- It seems like it would be interesting to compare a buffer based on bits of information rather than items -- using bits is popular both in visual memory studies and in psycholinguistics, and bit-vs-item based accounts make different predictions. Because the authors have a probabilistic model, it seems like they are in a good position to do this.

Author response:

We agree, this would be very interesting; we have now discussed this proposition in the Discussion. It would take some careful work to extend the implementation this way, but the present model indeed provides a good starting point for this.

Revised text:

[Discussion, l. 752-760]

The informational buffer capacity of the PPM-Decay model is currently defined as a fixed number of items, implying that each auditory event occupies an identically sized portion of auditory memory. However, it is possible that the auditory memory buffer is not restricted so much by the number of events but rather by the amount of information that these events represent. According to this proposition, highly expected or repetitive events would occupy less of the buffer, hence increasing its effective itemwise capacity (e.g. Watson & Foyle, 1985; Brady et al., 2009). It would be interesting to test a variant of the PPM-Decay model that implements such a buffer, perhaps quantifying the information carried by each event as its negative log probability according to the predictive model, after Shannon (1948).

***

Reviewer comment:

- It's worth thinking about some places where this model may not make the right predictions -- for instance, if you have a memorized piece of music or you tend to remember a particular style

of music for long periods of time. Is PPM-decay just an approximation to some fancier form of clustering genres?

Author response:

The original manuscript identified two domains where the PPM-Decay model might not make the right predictions:

- Sequences comprising continuous rather than discrete observations;
- Sequences comprising non-adjacent dependencies.

Following the suggestions of the reviewer, we have now added three more domains to this part of the manuscript:

- Situations where historic sequence statistics disappear for a while then come back later. This motivates the clustering ideas mentioned by the reviewer.
- Situations involving attention manipulation or sleep consolidation;
- Veridical memory, as opposed to schematic memory.

Revised text:

[See above for new paragraph on clustering methods, l. 733-745]

[Discussion, l. 746-751]

The memory-decay function in the PPM-Decay model currently only depends on the timepoints of event observations. However, one might expect memory retention to be influenced by various other phenomena, such as attention (Baker et al., 2004; Toro et al., 2005), or sleep consolidation (Durrant et al., 2011; Simon et al., 2017; Bianco et al., 2020). Though such phenomena are not currently captured by the PPM-Decay model, it should be possible to address them through various modifications to the memory-decay function without having to modify the wider modeling framework.

[Discussion, l. 761-770]

The PPM-Decay model addresses how listeners generate predictions based on a learned generative model of sequential structure: the resulting predictions may be termed 'schematic expectations'. However, given sufficient exposure to particular sequences, listeners may memorize the sequences themselves, allowing them to produce what may be termed 'veridical expectations'. While schematic expectations are relevant for individuals learning new languages and new musical styles, veridical expectations are relevant for individuals memorizing passages of literature or particular pieces of music. It seems that these two kinds of expectations may constitute distinct psychological mechanisms (e.g. Ockelford, 2006; Schubert & Pearce, 2015);

future work is required to understand how well the PPM-Decay model can extend past schematic expectations to veridical expectations.

***

Reviewer comment:

- What license is the software provided under?

Author response:

We have added a note about the MIT license to the main text.

Revised text:

[Introduction, l. 142-145]

We have developed an open-source implementation of the model in C++, made available in the R package *ppm* and released under the MIT license, that allows the user to configure and evaluate different variants of the PPM-Decay model on arbitrary sequences.

Response to Reviewer 2

Reviewer comment:

The manuscript presents an extension to the Prediction by Partial Matching (PPM) model, to formally include the notion of memory limitation. Three distinct tasks are described: the recovery of a time-varying statistical structure in artificially-generated sequences of symbols; the effect of exposure to predict the structure of musical scores; the prediction of behavioral performance in a regularity-detection task for a novel set of experimental data. In all cases, the core PPM idea successfully adapted to the task by adding some form of memory limitation. Part 1 shows that imposing an exponential decay on past information is beneficial to adapt to changing statistics. Part 2 introduces a non-zero asymptote to the decay and shows that this asymptote (representing long-term learning) has a different weight for predicting different musical genres. For part 3, a combination of a short, perfect memory store followed by exponential decay and noise is introduced, resembling some models of perceptual memory.

The manuscript provides a formal test of a natural extension to the PPM model. By adding memory limitations, the model is no more limited to evaluating the limit-case of an observer with perfect memory of the past, but has much greater flexibility to be fit to different tasks and situations. This is an important advance, which should be of clear interest to the readership of the journal. Even though subparts of the manuscript may at times feel like a paper within a

paper, there is still a logic in having all three studies in the same place. The fact that the precise form of memory limitations needed to fit the tasks turned out to be different in all three cases initially bothered me, but in part thanks to the Discussion, I am more convinced that this could be seen as a strength of the approach, as the model can be used as a tool to highlight specific aspects of memory involved in specific tasks.

I have very little comments with parts 1 and 2, which I found highly convincing. I thought that the artificial example of part 1 made the point clearly, and while results were perhaps unsurprising, the bigger idea that forgetting helps in changing environments is interesting. The conclusion reached in part 2, on differences of long-term vs short-term regularity with genre, would be of great interest to musicologists, and could have been the bulk of an independent manuscript in a different journal. I have a few more comments for part 3, with some issues that may need addressing prior to publication.

***

Reviewer comment:

L362. The motivation given to gather new experimental data (and not simply model Barascud et al. (2016)) is to test whether auditory memory is limited in terms of duration or capacity, citing 'historic' work supporting the capacity idea. I think this very interesting question is not limited to historic work, and furthermore that there may be yet other alternatives to describe the limits of auditory memory. For instance, the work of Demany and colleagues suggests almost perfect capacity and essentially no decay seconds in some tasks (Demany et al., 2008, Psych Science, see also the book chapter Demany and Semal 2007). Kang et al., 2017, J Acoust Soc Am, also outline alternative ideas based on randomly sampled pattern motifs. As the precise form of memory limitation is central to the argument made by the new task and subsequent modeling, maybe a more complete survey of the available alternatives would be useful?

Author response:

It makes sense to provide more context for the capacity limitation idea. Though there is not much directly relevant literature about this idea, we have now expanded this section to talk about more of the proximal literature, which should be helpful for contextualizing the reader.

Revised text:

[Experiment 3, l. 434-452]

The auditory sequences studied by Barascud et al. used very short cycle lengths, of the order of 1 s, and were therefore likely to fall within the bounds of echoic memory. Temporal limits to echoic memory are well-documented in the literature: echoic memory seems to persist for a few seconds, and is susceptible to interference from subsequent sounds (e.g. Winkler & Cowan,

2005). Informational limits to echoic memory are less well understood. The most relevant precedent to this paradigm in the literature seems to be Watson and Foyle (1985), studying change detection in sequences of pure tones where total sequence duration ranged from 60 to 2000 ms. In this regime, change-detection performance was largely unaffected by sequence duration, and was instead constrained by the number of tones in the sequence, pointing to an informational limit to echoic memory. However, related work has studied change detection where the tones were presented simultaneously rather than sequentially, and failed to find compelling evidence for memory capacity limits over and above sensory processing limits (Demany et al, 2008; Gregg et al, 2017). Another relevant precedent is Kang et al. (2017), who played participants pairs of adjacent click trains each of 1 s duration, and asked participants to answer whether the second click train was a repetition of the first click train. Performance decreased as click rate increased, which could reflect capacity limits in echoic memory, but could also reflect capacity limits in sensory processing. In summary, informational limits to echoic memory have received limited characterization in the literature, but nonetheless seem equally plausible to temporal limits as explanations of Barascud et al.'s results.

***

Reviewer comment:

L379. Remarkably for human behavior, but unfortunately for the manuscript's purpose, performance in the new task was very near ceiling for all conditions and participants. Doesn't this mean that there must exist a memory store of some kind that contained enough information for the task to be performed perfectly? Operationaly, would there be a statistics extracted from the PPM-decay model put forward that predicts perfect performance in all cases?

L382. As is commonly done in behavioral research, reaction times are considered as a surrogate measure of 'performance above ceiling'. So, RTs are chosen as the target for the modeling. However, I think this general idea needs a very careful justification here, as there may be other models/strategies that lead to different RTs without requiring any form of memory decay, thus defeating the main point of the modeling. For instance, what if it simply took longer to scan a perfect but longer memory trace?
I think what is missing for me is an explicit description of what it would take for an observer using the PPM-decay model to achieve both perfect performance for all conditions and slower RTs for some conditions. Such an explicit statement of the underlying assumptions could also help discussing possible alternative interpretations.

Author response:

In fact the ceiling performance is predicted by the model. This reflects the fact that once sufficient evidence has been accumulated as the sequence unfolds, the regularity automatically 'pops out' perceptually. The task manipulations change how long it takes for the participant to detect the change, but they do not prevent the participant from detecting the change eventually.

Therefore, RTs allow us to quantify the amount of sensory information (in terms of number of tones) that the listener needs to extract to detect the emergent regularity. We have updated the paragraph to make this point clearer.

It makes sense to discuss other potential explanations for the reaction-time effect. We have now added such a discussion at the end of this Experiment 3 section.

Revised text:

[Experiment 3, l. 465-471]

Response accuracy was close to ceiling performance across all conditions, with the exception of the condition with the maximum-duration cycles (20 tones each of length 75 ms), where some participants fell away from ceiling performance. This is consistent with Barascud et al. (2016); the task manipulations change how long it takes for the participant to detect the change, but they mostly do not prevent the participant from eventually detecting the change. Following Barascud et al. (2016), we therefore focus on interpreting reaction-time metrics (Fig 5B) rather than response accuracy.

[Experiment 3, l. 610-623]

Here we supposed that our observed trends in reaction times reflect auditory memory decay. It is worth considering alternative explanations for these trends. One possibility is that the trends reflect interference in auditory memory rather than memory decay *per se* (e.g. Brown, Neath, & Chater, 2007). This would be an important mechanistic distinction to make, but would not change our main conclusions about the importance of memory limitations for auditory prediction and the presence of a finite-capacity auditory memory buffer. A second possibility is that the reaction-time trends were not driven by memory decay, but were instead driven by older memories taking longer to retrieve from memory than newer memories. Likewise, this would be an important mechanistic distinction, but would not change our main conclusions. A third possibility is that the reaction-time trends were driven by sensory processing limitations. However, such limitations would predict that the faster tones be processed less effectively, meaning that more tones would be required for change detection in fast-tone conditions than in slow-tone conditions, which is not what we observe. Memory dynamics therefore still seem to provide the most plausible explanation for these effects.

***

Reviewer comment:

L398. The analysis uses differences of differences to make a discussion point. Why not use an ANOVA on the whole results and look for interactions?

Author response:

We've now reported the ANOVA analysis of that interaction as suggested.

Revised text:

[Experiment 3, l. 496-498]

This interaction between tone duration and cycle length was also evident from a 3 x 2 repeated-measures ANOVA ($F(2, 44) = 8.76$, eta squared = .07, $p < .001$).

***

Reviewer comment:

Fig 6. The proposed form of the decay function appended to the PPM is quite complex and implies four a priori choices: a perfect buffer of constant capacity that is fine-tuned to each stimulus duration; an abrupt drop at the end of this buffer; an exponential decay; additive noise. On the plus side, the logic for each refinement is well discussed in the text. However, one has to wonder how much of these choices were really constrained by the data. Would there be other, very different strategies that would fit the data? Or different parameter sets with the same model structure? This is I think important to ask, because if the modeling is to provide evidence for the nature of the underlying cognitive processes, one would want to know how unique and specific is a parameter set that happens to fit the task. The text mentions that the model was manually tuned, maybe some sort of parameter grid search would be useful?

Author response:

Yes, it is a good idea to give the reader a better idea of the model's parameter sensitivity. A comprehensive grid search proved computationally intractable, so instead we performed a perturbation analysis which we report now in the main text at the end of the 'Experiment 3' section. We have also added a passage to the Discussion discussing the possibility of alternative model configurations.

Revised text:

[Experiment 3, l. 591-609]

Finally, we performed a simple sensitivity analysis to understand how much the model's fit depended on the precise values of the model parameters. We took the five optimized model parameters (itemwise buffer capacity, buffer weight, half life, initial post-buffer weight, and retrieval noise), and represented each as a Gaussian with mean equal to its optimized value and with standard deviation equal to 15% of the mean. We then sampled 100 parameter vectors from

these Gaussians, reran the simulation with each of these parameter vectors, and compared the resulting model fits to that of the original parameter vector. We quantified model fit in terms of the consistency between mean model reaction times and mean participant reaction times, with reaction times measured in tones and aggregated by condition, and with consistency operationalized by two coefficients: the intraclass correlation coefficient (one-way, single measurement, absolute agreement), and the Spearman correlation coefficient. The former assesses how successfully the model predicts absolute reaction times, whereas the latter assesses how successfully the model predicts the order of reaction times. We found a mean intraclass correlation coefficient of .77 for the resampled parameters, compared to a coefficient of .96 for the original parameters, indicating that absolute model fit is moderately sensitive to the choice of parameters. In contrast, the mean Spearman correlation coefficient for the resampled parameters was essentially identical to that of the original parameters (rhos = .83, .83), indicating that the qualitative trends in the model predictions are comparatively robust to local changes in the parameters.

[...]

Now, this particular experiment only provided a limited amount of data with which to evaluate this relatively complex model, and it is difficult to dismiss the possibility of alternative model configurations predicting the data equally well. Nonetheless, our model provides a plausible hypothesis for how the observed response patterns came about, and our experiment provides a useful starting point for developing computational models that better resemble human auditory prediction.

***

Reviewer comment:

L 428: setting a buffer length of 15 items seems ad-hoc, given that the experiment tested sequences of 10 and 20 items and observed a drop in performance between the two. How confident are we that the value is not tied to the experimental details?

Author response:

It is true that the experimental data do not constrain buffer length very strongly, because we only collected data for cycle lengths of 10 and 20 items (note though that Figure 8 shows how the shorter buffer lengths of 5 and 10 do not work well for modeling the data). More generally, it seems likely that all of the decay kernel parameters will depend somewhat on the experimental task, even if the qualitative form is similar. We have therefore added a sentence to this passage highlighting the probable sensitivity to experimental details.

Revised text:

[Experiment 3, l. 525-528]

The precise parameters of this decay kernel come from manual optimization to the behavioral data, and it should be noted that these parameters may well be task-dependent; nonetheless, we will show that each qualitative component of the decay kernel seems to be necessary to explain the observed pattern of results.

***

Reviewer comment:

L439. The noise added to the model representation is described as having a sigma of 0.8. Is the unit the same as the weights, and if so, how to interpret such a value given that the maximum possible weight value is 1?

Author response:

We have now revised this passage to make the arithmetic clearer, and to emphasise that the noise is added to the total weight over all observations for a given n-gram, rather than being added to the weight for a single n-gram observation. This removes the potential confusion about there being a maximum weight value.

Revised text:

[Experiment 3, l. 533-538]

In our optimized model this noise component is implemented by sampling from a Gaussian with zero mean and standard deviation $s_e = 0.8$, taking the absolute value, and adding this to the total weight over all observations for a given *n*-gram. We avoid negative noise samples so as to avoid negative event counts, for which the PPM model is not well-defined.

***

Reviewer comment:

Methods: There were in fact more conditions run than described in main text, which is not great given that some conditions (CONT, STEP) were used as normalization for all the data presented. Without breaking the flow of the main story, I think it would be useful for the reader to form a clear picture of the task before delving into the methods.

Author response:

We have added a paragraph to the main text to provide this clarification.

Revised text:

[Experiment 3, l. 384-390]

Two simplified sequence types were also included for the purpose of baselining reaction times: 'CONT' sequences, which were simplified versions of RAND sequences that comprised just one repeated tone of a given frequency, and 'STEP' sequences, which were simplified versions of RAND-REG sequences where all tones within a given section had the same frequency, with this frequency differing between sections. We used the STEP trials to estimate baseline response times, and normalized the RAND-REG response times by subtracting these baseline response times (see *Methods* for more details).
